# Searching in the dark: Shining a light on some predictors of non-response to psychotherapy for borderline personality disorder

**DOI:** 10.1371/journal.pone.0255055

**Published:** 2021-07-27

**Authors:** Jane Woodbridge, Samantha Reis, Michelle L. Townsend, Lucy Hobby, Brin F. S. Grenyer

**Affiliations:** 1 Illawarra Health and Medical Research Institute and School of Psychology, University of Wollongong, Wollongong, Australia; 2 School of Education, Western Sydney University, Penrith, Australia; Medical University of Vienna, AUSTRIA

## Abstract

**Background:**

Borderline Personality Disorder (BPD) is a prevalent and serious mental health condition. People can experience recovery or remission after receiving psychotherapy for BPD; however, it is estimated that about 45% of people in well conducted treatment trials do not respond adequately to current psychological treatments.

**Aim:**

To further advance psychotherapies for BPD by identifying the factors that contribute to the problem of non-response.

**Method:**

184 consecutive participants with BPD in community treatment were naturalistically followed up over 12 months and measures of personality and social functioning were examined. Logistic regressions were used to determine which baseline factors were associated with the likelihood of being a non-responder after 12 months of psychotherapy. After 12 months, 48.4% of participants were classed as non-responders due to a lack of reduction in BPD symptoms according to the Reliable Change Index (RCI) method.

**Results:**

At baseline intake, patients who endorsed an adult preoccupied attachment relationship style and increased anger were more likely to be a non-responder regarding BPD symptoms at 12 months. In addition, those with preoccupied attachment patterns in their adult relationships were more likely to be non-responders regarding general psychological distress at follow up. Higher baseline levels of paranoia and endorsement of a dismissive adult relationship style was associated with being a non-responder in regard to global functioning.

**Conclusions:**

Consistent with previous research, almost half of the sample did not achieve reliable change at 12-month follow up. A relationship style characterised by preoccupied insecurity and high anger seemed to be particularly challenging in being able to benefit from psychotherapy. This style may have affected both relationships outside, but also inside therapy, complicating treatment engagement and alliance with the therapist. Early identification and modification of treatment based on challenges from these relationship styles may be one way to improve psychotherapy outcomes for BPD.

## Introduction

Despite borderline personality disorder (BPD) being a severe and high prevalence mental health disorder [1], research has demonstrated that many people with BPD can be effectively treated with psychotherapy leading to remission and better functioning [2, 3]. Over the last two decades, numerous outcome studies have demonstrated the efficacy of various specialised psychotherapies for treating BPD [2, 4]. Despite these findings, no treatment to date has shown universal effectiveness, meaning a portion of people do not improve. Improvement is often understood as a person no longer meeting criteria for BPD, or reliably decreasing their scores on BPD specific measures after treatment. For example, published studies have variously reported: 30 to 41.6% of participant samples did not respond to Dialectical Behaviour Therapy (DBT; [5–7]; 6 to 81.3% did not respond to Schema Focused Therapy (SFT; [8–11]; 48.8 to 57.1% did not respond to Transference Focused Therapy (TFP; [10, 12]; 58.8% did not respond to Cognitive Analytic Therapy [13]; and 48% did not respond to Mentalisation Based Therapy (MBT; [14]. Non-response among the samples in these studies ranged from 6% to 81.3% with an average of about 45%. Additionally, investigating across studies has demonstrated that overall treatment effects for BPD specific psychotherapies remain small (*g =* 0.34; 95% CI, 0.15–0.53 for DBT and *g =* 0.41; 95% CI, 0.12–0.69 for psychodynamic therapies), and are unstable at follow up [4]. These results illustrate the need to continue working to further improve the available psychotherapies.

Manualised evidenced-based specialised psychotherapies are limited in availability to the majority of people with BPD [15, 16]. This is likely due to the high ratio of people with BPD seeking treatment to clinicians certified to implement such treatments [16], alongside the intensity of the training requirements, and the time it takes for clinicians to become certified [17]. Therefore, many people with BPD receive non-manualised, unspecialised, uncontrolled treatments in the community [15, 16]. This treatment approach is typically termed “treatment as usual” (TAU) and is often used as a control arm in treatment outcome studies of manualised treatments to represent the kind of treatment available to people with BPD. TAU is typically heterogenous, difficult to define or measure, and has previously been thought of as ineffective and even iatrogenic [18]. However, meta-analyses demonstrate that people treated with TAU experience small to moderate improvements in BPD symptoms, general psychopathology and functioning, with these benefits further increasing with the amount of time engaged in treatment [18]. Due to TAU being more readily available, and to have demonstrated efficacy, it is appropriate to test for factors associated with non-response within this real-world approach.

Non-response is a well-known phenomenon in psychiatry. Meta-analyses of psychotherapy outcome studies have found that the absolute efficacy remains at a consistent effect size of .80 across time and studies [19]. These statistics are taken to demonstrate the effectiveness of psychotherapy. However, if the alternative is considered, the same statistics suggest that for every three people being treated with psychotherapy, two of those will not have a better outcome. Large scale reviews using individual statistics have reported that 67.2% [20] and 50.1% [21] of patients with high prevalence disorders (e.g. depression, anxiety) reliably improve after an average of 12.7 and 8.5 sessions of psychotherapy respectively. This suggests that between 32.8% and 49.9% of patients are not responding to psychotherapy, which highlights the importance of continuing efforts to improve psychotherapy outcomes. Investigating non-response in BPD specifically is crucial due to the significant distress and functional difficulties, high rates of suicide, extensive use of mental health resources and an elevated risk of relapse associated with the disorder [22–25]. In addition, research suggests that it can be challenging for clinicians to recognise which particular clients are not responding to treatment before termination [26–28]. Typically, clinicians do not as yet have established criteria upon which to make to predictions about prognosis or systematically guide treatment planning to prevent non-response. Therefore, research into non-response to psychotherapy has the potential to improve response to psychotherapy and clinician actions.

Criterion A in the DSM-5 Alternative Model of Personality Disorders describes the core of personality psychopathology to be disturbances in self and other (interpersonal) functioning [29]. Self-functioning pertains to identity, which incorporates stability of self-esteem and accuracy of self-appraisal; and self-direction which incorporates utilisation of constructive internal standards and the ability to self-reflect productively. We suggest that being high in self-criticism may indicate disturbances in all of these elements of self-functioning. Interpersonal functioning pertains to empathy and intimacy. Adult attachment relational styles capture some elements of interpersonal functioning and perhaps the intersections between self-other models of interpersonal security-insecurity. Attachment and self-criticism have previously been associated with the development, maintenance and experience of BPD [30, 31] and with psychotherapy outcomes [32, 33]. Therefore, it is important to understand how these factors may contribute to differential outcomes from psychotherapy for individuals with BPD.

In the context of BPD, adult attachment relationship style has long been implicated in its aetiology, conceptualisation and treatment [34–37]. The core symptoms and interpersonal difficulties experienced by people with BPD are increasingly being understood as arising from impairments in the underlying attachment relationship organisation [30, 37]. Accordingly, addressing core relationship difficulties is fundamental in the treatment of BPD [35, 38]. Attachment theory may also offer an explanation for treatment non-response among people with BPD. Insecure attachment styles comprise negative internal representations of the self and others, and imbalances in under and over reliance on self and others [39, 40]. This can culminate in difficulties with trusting others, impeding the development of healthy therapeutic alliances with clinicians and, in turn, contributing to difficulties in achieving good outcomes from treatment [33, 37, 41]. A recent meta-analysis supports that insecure attachment styles (fearful, preoccupied and dismissive) are related to poorer psychotherapy outcomes, and secure attachment is related to better outcomes [33] Therefore, it follows that insecure attachment styles; namely, fearful, preoccupied and dismissive, could contribute to non-response to psychotherapy for people with BPD.

Self-criticism, along with self-loathing, inferiority and shame, is prevalent among people with BPD [42] and considered central to the experience of the disorder [31]. Self-criticism involves excessively punitive self-evaluation, self-depreciation, unrealistic goal setting and a perpetual perception of failure [43, 44]. In the context of BPD, self-criticism has been found to be related to identity disturbances and self-destructive behaviours [45]. People with BPD may, for example, use self-harm to regulate their emotions due to their severe self-critical beliefs that they are bad and deserving of punishment. This notion is supported by research that found self-criticism strongly predicts higher willingness to endure pain among self-injuring people [46]. Self-criticism is associated with negative outcomes and poor treatment response in general [32, 47], and for people with BPD specifically [48]. Therefore, in the context of BPD, it is likely that higher levels of self-criticism may contribute to non-response to psychotherapy.

Previous attempts have been made to understand the effects of individual BPD symptoms on treatment outcome; however, the majority of this work has sought to identify factors that facilitate recovery as opposed to factors that act as barriers to recovery. Furthermore, the literature often returns mixed and contrary results. For instance, self-harm and suicidality have been reported to have positive [49], poor [50], mixed [51] and no associations [52] with therapeutic outcomes. Dissociation has been found to predict both poor response [53] and positive response [54] regarding general psychopathology after receiving DBT, to have no impact on remission from self-harm [55] nor any relationship with therapeutic outcomes [52]. Impulsivity has been associated with poor vocational and psychosocial outcomes [56–58], strong re-increases of symptom severity between treatment cessation and follow up [59] and improvements in anger expression after therapy [52]. Emotional instability has been found to be related to positive response to therapy [51], poor functioning after therapy [60] and to have no predictive relationship with therapeutic outcomes [52]. A systematic review of prospective predictors of positive outcomes (treatment response) after psychotherapy for BPD [61] found firstly, no consistent relationship between sociodemographic variables or pre-treatment comorbidities and outcomes. Secondly, that research has produced mixed results regarding self-harm and psychotropic medication use.

This disparate array of research was collected from multiple types of studies, of which some were experimental, some correlational and some observational. Some authors found that their results directly contradicted their hypotheses [52]. Furthermore, the outcomes employed were also highly varied (i.e., general psychopathology, self-harming behaviours, reductions in the same criteria, psychosocial and vocational functioning), while very few investigated the relationship between each criteria and response to therapy in terms of symptomatic remission from BPD. Moreover, the inconsistencies of the findings further highlight the need to directly investigate any associations between specific BPD criteria and non-response. Due to the inconsistent findings to date, no hypotheses regarding which specific BPD criteria will relate to non-response can be made in the present investigation. Rather, an exploratory approach including all individual symptoms of BPD (i.e., relationship instability, suicidal ideation, impulsivity, emotional instability, anger, paranoia, dissociation, chronic emptiness, identity disturbance and abandonment sensitivity) in the analyses will be undertaken.

We therefore proposed a study to investigate not only those factors associated with the characteristic difficulties of people with BPD but also to investigate a "self" identity factor (self-criticism) and an "other" relationship factor (adult attachment relationship style) to more broadly understand how the core features of personality disorder may contribute to non-response.

## Method

### Participants and procedure

Adult patients with diagnosed with BPD seeking care through public mental health services were prospectively and consecutively invited to participate. Exclusion criteria were: evidence of psychosis, alcohol or other drug disorder as the primary presenting problem rather than BPD and/or imminent level of risk determined by the relevant Mental Health Triage Policy. Clinical diagnosis of BPD was confirmed by trained mental health practitioners using a structured interview protocol of mental health outcomes and assessment, the NSW Mental Health Outcomes and Assessment Tools [62]. All consenting participants gave written informed consent following approval from the University of Wollongong & Illawarra Shoalhaven Local Health District Health and Medical Human Research Ethics Committee. At intake, participants were interviewed and completed a broad set of measures relevant to possible prognostic factors related to improvement. No data was collected on people who declined to participate in the study. Participants were then followed-up after 12 months and were re-interviewed.

A total of 379 participants agreed to enter the study; however, 182 did not participate in the follow up interview. Participants who did not complete the follow up interview were younger (*M* = 30.16, *SD* = 1.27), compared to those who did complete the follow up interview (*M* = 34.61, *SD* = 14.20), *t*(349.53) = -3.272, p = .001, 95% CI [-7.11, -1.77], *d* = .34. However, neither gender χ^2^ (1, *N* = 369) = .872, *p* = .352, φ = .049, nor relationship status χ^2^ (2, *N* = 289) = 1.253, *p* = .534, φ = .066, was significantly related to non-completion. A further 13 participants were excluded from analyses due to no longer meeting criteria for BPD during their first data collection interview. Therefore, 184 participants with intake and follow up data were analysed in the present study. For all patients, treatment provided was psychological therapy following a stepped care model [63]. This stepped care model was founded in recommendations from clinical guidelines for BPD [63–65], which has been previously described elsewhere [66–68]. At intake, a brief intervention was first offered as part of the stepped care model between acute crisis presentation and longer-term treatments. The brief intervention consisted of one month of outpatient stabilisation and included connection with the patient’s family or carer if possible. Participants then engaged in evidence-based treatment as usual (TAU) with providers in the community [63] as clinically indicated. Due to the treatment being TAU, treatment dropout was allowed to vary freely and was not monitored, as all participants were invited to participate in follow up interviews irrespective of their clinical status or whether they remained engaged in treatment. At 12-month follow up most participants (81.2%) were still engaged in psychological therapy. In addition, 43% were consulting a treating psychiatrist and 9.7% were attending a support group.

### Measures

#### BPD symptoms

Interviewers asked participants to rate each of the DSM-5 [29] BPD criteria problem area for its current severity over the past two weeks (1 = *none of the time* to 6 = *all of the time*) in order to measure BPD symptomology (Miller et al., 2018). We asked two questions in relation to DSM-5 BPD Criterion 9; a question about distrust (paranoia), and a question about things feeling unreal (dissociative experiences), a convention also adopted in the McLean Screening Instrument for BPD method [69]. Symptom scores thus ranged from 10 to 60, providing a dimensional understanding of BPD symptoms [70, 71]. This method has previously demonstrated good internal consistency and predictive validity [56]. Reliability for the measure of BPD symptoms in the present study was α = .819.

#### Adult attachment relationship style

The Relationship Questionnaire (RQ; [39] was employed to measure current adult attachment relationship style based on a four-category attachment prototype model. The RQ comprises four short paragraphs describing each attachment prototype; secure, preoccupied, fearful and dismissing (e.g., preoccupied attachment: “I want to be completely emotionally intimate with others, but I often find that others are reluctant to get as close as I would like. I am uncomfortable being without close relationships, but I sometimes worry that others don’t value me as much as I value them”). Both the continuous and categorical versions of the measure were used. Participants rated how much each paragraph described them from 0 (*not at all like me*) to 100 (*very much like me*) to generate a continuous rating on each of the four categories. Furthermore, participants selected which prototype described them best to obtain a categorical preference. Previous research has demonstrated both convergent and discriminant validity of the RQ through correlations in the expected directions with concordant attachment types from other measures of attachment [39, 72, 73].

#### Self-criticism

The Depressive Experiences Questionnaire—Self-Criticism scale (DEQ-SC-6; [74] was used to measure trait self-criticism defined as excessively harsh judgements of the self with a constant sense of failure. Participants responded to 6 items on a 7-point Likert Scale (1 = *strongly disagree* to 7 = *strongly agree*) to obtain a total ranging between 6 to 42. Items include “I tend to be very self-critical”. Previous research has reported reliabilities for the DEQ-SC-6 ranging from α = .66 to .84, while validity has been demonstrated through strong correlations with other measures of self-criticism [74]. Reliability in the present study was α = .739.

#### Psychological distress

The Mental Health Inventory-5 (MHI-5; [75] is a five-item questionnaire from the Short Form-36 (SF-36) used to assess severity of psychopathological distress. Each question assesses one aspect of mental health: depression, anxiety, positive affect, loss of behavioural/emotional control and psychological wellbeing (e.g., anxiety: “Have you been a very nervous person”). Participants responded on a 6-point Likert scale (1 = *none of the time* to 6 = *all of the time*) to obtain a total range from 6 to 30. The MHI-5 has demonstrated excellent sensitivity for detecting mood and anxiety disorders and has comparable performance with longer measures [75, 76]. Previous research has demonstrated the validity of the measure through correlations with other measures of depression [76] and reliability with α = .74 [77] to α = .83 [78]. Reliability in the present study was α = .792.

#### Functioning

The Global Assessment of Functioning (GAF; [79] is a widely used single-item, clinician rated assessment of social and occupational functioning independent of psychological symptoms. The GAF was assessed at 12 month follow-up only. The scale ranges from 0 to 100 where the 1 to 10 bracket is “*Persistent danger of hurting self or others (e*.*g*. *recurrent violence) or persistent inability to maintain minimal personal hygiene or serious suicidal act with clear expectation of death*” and the 91 to 100 bracket is “*Superior functioning in a wide range of activities*, *life’s problems never seem to get out of hand*, *is sought out by others because of his or her many positive qualities*. *No symptoms*”. Previous research has demonstrated the GAF is a valid measure for rating social and occupational functioning and has high inter-rater reliability [80].

## Data analysis

All analyses were conducted using IBM SPSS Statistics, Version 25.0 software [81]. Data cleaning was conducted prior to analysis. No outliers were found; however, some violations of normality were detected via significant skewness and kurtosis values. However, these values can be significant in large samples even when violations of normality are mild [82]. Furthermore, visual inspections of the main outcome variables revealed positive skew, which is to be expected in a clinical sample [82]. Moreover, the relatively large sample, the use of bias corrected accelerated bootstrapped confidence intervals and robust regression analyses protect against potential bias [82]. Missing values analysis indicated that data were missing completely at random (Little’s missing completely at random χ^2^ = 1506.722, *p* = 1.000). Based on this finding, expectation maximisation (EM) was used to impute missing data as recommended by Tabachnick and Fidell [83] and Schafer and Graham [84].

Participants were categorised as ‘non-responders’ via Jacobson and Truax’s [85] method for calculating reliable change. This method allows the calculation of the magnitude of change, while taking into account the error of measurement, such that it can be relied on to be statistically significant [85, 86]. The Reliable Change Index (RCI) method is widely used in the literature for computing change scores [87–91]. The formula for calculating the RCI is as follows:

$$RCI=\frac{X_{pre}-X_{post}}{S_{diff}},\;\mathrm{where}\;S_{diff}=\sqrt{2\cdot {\left(SE\right)}^{2}}\;\mathrm{and}SE=SD\cdot \sqrt{1-r_{tt}}$$

*X*_pre_ = BPD symptom total of the continuous severity score at baseline

*X*_post_ = BPD total of the continuous score at 9-month follow up

*SD* = Standard deviation of BPD baseline score

*r_tt_* = Reliability of the BPD (Cronbach’s alpha in this case)

Whilst most studies employing the RCI often obtain norms for the outcome measures used from clinical populations, the standard deviation and reliability values from the study data can be employed to calculate the RCI where such norms are not available [85, 91, 92]. Given that the norms for the measure of BPD symptoms used in the current study were not available, the standard deviation and reliability values from the current sample at baseline were used to calculate the RCI (see values in Table 1).

**Table 1:** Standard deviations and reliability values for calculating reliable change indices.

Measure	Standard Deviation	Reliability Value
BPD	9.724	.819
MHI-5	4.568	.792

The RCI was calculated to create categorical variables to discriminate ‘non-responders’ (those who had not changed i.e., remained symptomatic) from ‘responders’ (those who had reliably changed for the better i.e., less symptomatic). Participants were considered ‘responders’ if they received an RCI score of 1.96 and above (greater change in the direction of improvement i.e., less symptoms), which corresponds to a 95% confidence interval [91]. The GAF was also used to create a categorical variable pertaining to overall functioning including symptoms, social and occupational functioning. Participants who scored 71 and above at 12-months were considered to be ‘functional’, while those who scored 70 and below were considered to be ‘less-functional’ as defined in previous studies [3].

The categorical variables (‘non-responders’ vs ‘responders’) were used as DVs in three logistic regressions to explore which factors were related to the likelihood of being a ‘non-responder’: (1) non-response in terms of BPD symptomology; (2) non-response in terms of psychological distress as measured by the MHI-5; and (3) non-response in terms of global functioning as measured by the GAF. The factors explored for their contribution to non-response were the individual BPD symptoms (relationship instability, suicide/self-harm, impulsivity, emotional instability, anger, paranoia, dissociation, chronic emptiness, identity disturbance, and abandonment sensitivity), self-criticism and the four attachment styles (secure, fearful, preoccupied and dismissive). These were entered into the analyses as IVs.

To determine whether demographic variables should be included in the main analyses as covariates, *t* tests and chi square tests were conducted. Age was not significantly different between responders (*M* = 33.65, *SE* = 1.52) and non-responders (*M* = 34.14, *SE* = 1.44), *t*(178) = -.231, *p* = .818, BCa 95% CI [-4.62, 3.65], *d* = 0.03, regarding BPD symptoms. Furthermore, gender χ^2^ (1, *N* = 183) = .149, *p* = .699, φ = -.029 and relationship status χ^2^ (1, *N* = 148) = 1.569, *p* = .210, φ = .103 were not significantly different between groups. Therefore, age, gender and relationship status were excluded from subsequent analyses.

## Results

The analysed sample consisted of 184 participants who ranged in age from 18 to 68. Sample characteristics can be seen in Table 2.

**Table 2:** Sample characteristics of people with Borderline Personality Disorder (N = 184) followed up for 12-months.

	M	SD	Percentage	N
**Demographics at baseline**				
Age	33.89	14.02		
Gender				
Female			73.9%	136
Male			25.5%	47
Missing			0.5%	1
Relationship Status				
In a relationship			17.9%	33
Single			62.5%	115
Missing			19.6%	36
**Clinical characteristics at baseline**				
Met criteria for BPD			100%	184
BPD symptom severity total (continuous 10–60)	36.03	8.59		
MHI-5 Psychological Distress Total (5–30)	21.49	4.23		
DEQ Self-Criticism Total (6–48)	32.05	6.57		
Attachment				
Secure			11.41%	19
Fearful			37.5%	69
Preoccupied			31.52%	58
Dismissing			19.57%	36
**Clinical characteristics at follow up**				
Met criteria for BPD			69.6%	128
BPD symptom severity total (continuous 10–60)	25.18	10.21		
MHI-5 Psychological Distress Total (5–30)	16.08	5.90		
GAF Functioning (0–100)	58.29	16.72		
	Min = 0	Max = 90		

Note: BPD = Borderline Personality Disorder; MHI-5 = Mental Health Inventory-5; DEQ = Depressive Experiences Questionnaire; GAF = Global Assessment of Functioning (12 month follow-up only).

In the sample, *n* = 95 (51.6%) participants demonstrated sufficient change in BPD symptoms according to their RCI scores after treatment to be categorised ‘responders’, while *n* = 85 (48.4%) did not, and were thus classified as ‘non-responders’. There were *n* = 5 (2.7%) whose RCI score was -1.96 or below, suggesting reliable deterioration (i.e. more symptomatic at follow up than baseline). Considering the small proportion of people who reliably deteriorated, they were included in the ‘non-responders’ group. Subsequent analyses were conducted using ‘responders’ and ‘non-responders’ as the grouping variable for comparison. Percentages of non-response vs response, with means (M) and standard deviations (SD) for the three measures can be found in Table 3.

**Table 3:** Responder and non-responder estimates by Reliable Change Index for BPD symptom severity, psychological distress, and global functioning.

Non-response Type	Non-responders	Responders
	N (%)	N (%)
	M (SD)	M (SD)
BPD symptom severity	89 (48.4%)	95 (51.6%)
	32.67 (8.78)	18.17 (5.27)
Psychological Distress (MHI-5)	92 (50%)	92 (50%)
	19.9 (5.01)	12.2 (3.77)
Global Functioning (GAF)	147 (80.3%)	36 (19.7%)
	53.3 (14.8)	78.27 (5.20)

### Non-response to treatment regarding BPD symptoms

A logistic regression analysis was used to explore which factors were related to being classified as a ‘non-responder’ in regard to BPD symptom severity after treatment. The predictor variables were the individual symptoms of BPD, self-criticism and the four attachment styles. The Hosmer and Lemeshow test demonstrated that the model fit the data well, χ^2^ (8, *N* = 184) = 6.99, *p* = .537. The model could correctly classify 60.7% of non-responders and 68.4% of responders, with an overall success rate of 64.7%. Table 4 displays the results from the logistic regression.

**Table 4:** Individual regression coefficients (b), standard errors (SE), Wald chi-square statistics (Wald χ^2^), Odds Ratios (OR) with 95% confidence intervals [95%CI], and p values for each individual predictor for the likelihood of being categorised as a ‘non-responder’ in regard to BPD symptom severity after treatment.

Predictor Variable	*b*	SE	Wald χ^2^	OR [95% CI]	*p*
Constant	-.460	1.082	.181	.631	.671
BPD Relationship Instability	-.042	.117	.131	.959 [.762, 1.205]	.718
BPD Suicide/Self-Harm	-.075	.123	.373	.928 [.729, 1.180]	.541
BPD Impulsivity	.029	.141	.043	1.030 [.781, 1.358]	.835
BPD Emotional Instability	-.254	.168	2.270	.776 [.558, 1.079]	.132
BPD Anger	.353*	.165	4.559	1.424 [1.029, 1.969]	.033
BPD Paranoia	-.174	.131	1.753	.840 [.650, 1.087]	.186
BPD Dissociation	-.259	.135	3.703	.772 [.593, 1.005]	.054
BPD Chronic Emptiness	-.009	.165	.003	.991 [.717, 1.369]	.955
BPD Identity Disturbance	.220	.118	3.492	1.247 [.989, 1.571]	.062
BPD Abandonment Sensitivity	-.070	.107	.427	.933 [.757, 1.149]	.513
DEQ Self-Criticism	.026	.028	.857	1.027 [.971, 1.085]	.355
RQ Secure Attachment	-.006	.007	.759	.994 [.982, 1.007]	.384
RQ Fearful Attachment	.002	.006	.074	1.002 [.989, 1.015]	.785
RQ Preoccupied Attachment	.013*	.006	4.415	1.013 [1.001, 1.025]	.036
RQ Dismissive Attachment	-.001	.005	.025	.999 [.989, 1,010]	.874

*Note*.

* *p* < .05 BPD = Borderline personality disorder symptom; DEQ = Depressive Experiences Questionnaire; RQ = Relationship Questionnaire.

Preoccupied attachment style (*b* = .013, *p* = .036) and anger (*b* = .353, *p* = .033) were significantly associated with non-response to treatment regarding BPD symptom severity. The corresponding odds ratio for anger of 1.424 indicated that while holding all other variables constant, a one unit increase in log-transformed anger made it 1.424 times or 42.4% more likely that a person would not respond to treatment. For preoccupied attachment style, the corresponding odds ratio of 1.013 demonstrated that a one unit increase in log-transformed preoccupied attachment style ratings made it 1.013 times more likely that a person would be classified as a ‘non-responder’ based on BPD symptomology. Specifically, participants had a 1.3% increase in risk of unchanged or higher BPD symptomatology after treatment for each one unit increase in preoccupied attachment scores (0 to 100).

### Non-response to treatment regarding psychological distress

A second logistic regression analysis was used to explore which factors were associated with being classified as a ‘non-responder’ regarding psychological distress as measured by the MHI-5. The predictor variables were the individual symptoms of BPD, self-criticism and the four attachment styles. The Hosmer and Lemeshow test demonstrated that the model fit the data well, χ^2^ (8, *N* = 184) = 2.94, *p* = .938. The model could correctly classify 65.2% of non-responders and 66.3% of responders, with an overall correct classification rate of 65.8%. Table 5 displays the results from the logistic regression.

**Table 5:** Individual regression coefficients (b), standard errors (SE), Wald chi-square statistics (Wald χ^2^), Odds Ratios (OR) with 95% confidence intervals [95%CI], and p values for each individual predictor for the likelihood of being categorised as a non-responder regarding psychological distress after treatment.

Predictor Variable	*b*	SE	Wald χ^2^	OR [95% CI]	*p*
Constant	.933	1.102	.716	2.541	.398
BPD Relationship Instability	-.050	.117	.183	.951 [.756, 1.196]	.669
BPD Suicide/Self-Harm	-.092	.123	.560	.912 [.718, 1.160]	.454
BPD Impulsivity	.064	.140	.208	1.066 [.810, 1.403]	.648
BPD Emotional Instability	-.236	.167	1.993	.790 [.570, 1.096]	.158
BPD Anger	-.036	.161	.050	.965 [.704, 1.332]	.823
BPD Paranoia	.058	.129	.202	1.060 [.822, 1.366]	.653
BPD Dissociation	-.197	.133	2.199	.821 [.633, 1.056]	.138
BPD Chronic Emptiness	-.211	167	1.602	.810 [.584, 1.123]	.206
BPD Identity Disturbance	.027	.117	.055	1.028 [.879, 1.332]	.814
BPD Abandonment Sensitivity	.079	.106	.548	1.082 [.879, 1.332]	.459
DEQ Self-Criticism	.003	.028	.011	1.003 [.949, 1.060]	.915
RQ Secure Attachment	-.002	.007	.072	.998 [.985, 1.011]	.788
RQ Fearful Attachment	.003	.007	.271	1.003 [.991, 1.016]	.603
RQ Preoccupied Attachment	.015*	.006	5.559	1.015 [1.002, 1.027]	.018
RQ Dismissive Attachment	.004	.005	.455	1.004 [.993, 1.014]	.500

Note.

* *p* < .05 BPD = Borderline personality disorder symptom; DEQ = Depressive Experiences Questionnaire; RQ = Relationship Questionnaire.

Preoccupied attachment (*b* = .015, *p* = .018) was the only significant factor related to non-response regarding psychological distress severity. The corresponding odds ratio of 1.015 indicated that while holding all other variables constant, a one unit increase in log-transformed preoccupied attachment style made it 1.015 times more likely that a participant would be classified as a non-responder based on their psychological distress score. Participants had a 1.5% increase in risk that their psychological distress would be unchanged or more negative after treatment for each one unit increase in their preoccupied attachment score (0 to 100).

### Non-response to treatment regarding global functioning

A third logistic regression analysis was used to explore which factors were associated with being classified as a ‘non-responder’ in terms of functionality as measured by the GAF. The predictor variables were the individual symptoms of BPD, self-criticism and the four attachment styles. The Hosmer and Lemeshow test demonstrated that the model fit the data well, χ^2^ (8, *N* = 184) = 10.18, *p* = .253. The model could correctly classify 97.3% of non-functional participants and 8.3% of the functional participants, with an overall success rate of 79.8%. Table 6 displays the results from the logistic regression.

**Table 6:** Individual regression coefficients (b), standard errors (SE), Wald chi-square statistics (Wald χ^2^), Odds Ratios (OR) with 95% confidence intervals [95%CI], and p values for each individual predictor for the likelihood of being categorised as ‘less-functional’ after treatment.

Predictor Variable	*b*	SE	Wald χ^2^	OR [95% CI]	*p*
Constant	.033	1.360	.001	1.034	.980
BPD Relationship Instability	-.397*	.173	5.269	.672 [.479, .944]	.022
BPD Suicide/Self-Harm	.325	.171	3.621	1.383 [.990, 1.933]	.057
BPD Impulsivity	.256	.178	2.077	1.292 [.912, 1.831]	.150
BPD Emotional Instability	-.356	.230	2.389	.701 [.446, 1.100]	.122
BPD Anger	.010	.219	.002	1.010 [.658, 1.552]	.962
BPD Paranoia	.370*	.170	4.746	1.448 [1.038, 2.021]	.029
BPD Dissociation	-.017	.172	.009	.983 [.702, 1.378]	.923
BPD Chronic Emptiness	.197	.203	.942	1.218 [.818, 1.813]	.332
BPD Identity Disturbance	.009	.150	.004	1.009 [.752, 1.354]	.950
BPD Abandonment Sensitivity	.121	.147	.678	1.128 [.847, 1.501]	.410
DEQ Self-Criticism	-.017	.036	.208	.984 [.916, 1.056]	.648
RQ Secure Attachment	-.011	.009	1.448	.989 [.972, 1.007]	.229
RQ Fearful Attachment	-.005	.009	.392	.995 [.978, 1.012]	.531
RQ Preoccupied Attachment	.006	.008	.498	1.006 [.990, 1.023]	.481
RQ Dismissive Attachment	.018*	.008	5.937	1.018 [1.004, 1.034]	.015

*Note*.

* *p* < .05 BPD = Borderline personality disorder symptom; DEQ = Depressive Experiences Questionnaire; RQ = Relationship Questionnaire.

Relationship instability (*b* = -.397, *p* = .022), paranoia (*b* = .370, *p* = .029), and dismissive attachment style (*b* = .018, *p* = .015) were all significantly associated with non-response regarding functionality.

The corresponding odds ratio for relationship instability of .672 indicated that while holding all other variables constant, a one unit increase in log-transformed relationship instability made it 1.328 times less likely that a person would be low-functioning after treatment. Specifically, participants had a 32.8% reduction in risk that they would be classified as low functioning after treatment for each one unit increase on their relationship instability. The corresponding odds ratio for paranoia of 1.448 indicated that while holding all other variables constant, a one unit increase in log-transformed paranoia made it 1.448 times or 44.8% more likely that a participant would be non-functional. The corresponding odds ratio of 1.018 for dismissive attachment indicated that while holding all other variables constant, a one unit increase in log-transformed dismissive attachment style made it 1.018 times more likely that a participant would be non-functional following treatment. Participants had a 1.8% increase in risk that they would be non-functional after treatment for each one unit increase on their dismissive attachment rating (0 to 100).

## Discussion

The present study examined the widespread problem of non-response to psychological therapy for BPD by following 187 patients in treatment for 12 months. An exploratory approach was taken regarding individual BPD symptoms and other self (self-criticism) and interpersonal factors (adult attachment relationship style) considering the inconsistency of findings from previous research.

In the current sample, 48.4% of participants were classified as ’non-responders’ using the RCI estimate on BPD symptom changes. Previous studies have reported that, on average, 45% of their samples did not respond to treatment [5–14], which is demonstrably similar to the 48.4% that did not respond in the current study. While roughly half of the present sample achieved reliable reductions in BPD symptom severity (as measured by a reduction of number of criteria met), 80.3% remained less functional (with a score of 70 and below) on the GAF. This result reiterates previous research which has reported that although symptomatic remission is readily achievable, regaining functionality is particularly challenging for people with a lived experience of BPD [93–96].

Results indicated that an adult preoccupied attachment relationship style and higher anger at treatment entry was associated with a higher risk of being a non-responder in terms of BPD symptom severity at 12 months. Preoccupied attachment style alone was associated with higher risk of being a non-responder in terms of psychological distress. A dismissive attachment style and higher paranoia was associated with a higher risk of being a non-responder in terms of global functioning, while higher relationship instability was associated with lower risk of being a non-responder in terms of functioning. No significant results were found for secure attachment, fearful attachment or self-criticism.

An adult preoccupied attachment style of relating was particularly associated with being a non-responder to BPD treatment. This finding is consistent with Strauss et al. [97] who found that an ambivalent-preoccupied attachment style was associated with the least favourable outcomes after therapy. People with preoccupied attachment styles in other studies have previously been noted to be difficult to treat [33], to show less improvement [98], to take longer to show behavioural changes [33] and to simultaneously seek contact and resist help [99].

An adult preoccupied attachment relationship style ascribes a negative perception of self and a positive perception of others. This can lead to having a low self-worth that is dependent on the approval of others [33, 39, 100]. Idealising others, while devaluing oneself, could promote a sense of hopelessness and dependency on others. Previous research has found associations between hopelessness and increased efforts to avoid abandonment, factors likely to be high in those with a preoccupied attachment style [52]. In the present study, abandonment sensitivity and preoccupied attachment style were significantly correlated as expected, given the overlap in these constructs. Hopelessness could contribute to a lack of self-efficacy, motivation and responsibility for one’s own recovery, especially if the person believes other people hold the power for recovery, which is likely in the context of an adult preoccupied attachment relationship style. Accordingly, therapists have been encouraged to be careful not to be overly caring or directive towards a patient with an adult preoccupied attachment relationship style, but rather to assist them to learn how to develop their own efficacy and agency [100, 101].

Mentalising difficulties have been found to be common to people with adult preoccupied attachment relationship styles [102]. People with preoccupied attachments have often experienced inconsistent caregiving or abuse in their childhoods [103] with such experiences fostering excessive relational vigilance, which is known to be prohibitive to developing healthy mentalising capacities [104]. This means that it can be extremely difficult for people with adult preoccupied attachment relationship styles to anticipate, identify, understand or link their internal experiences to their subsequent behaviours. Furthermore, people with preoccupied attachment styles can struggle to engage in perspective taking, especially in periods of distress [35]. These deficits could lead to an inability to engage in flexible thinking or developing insights into the self [103, 105], further hindering opportunities for behavioural change. All of these factors could lead to difficulties for those with BPD in responding to psychological treatment, considering that therapy often requires an understanding of emotions in self and others, an understanding of the role emotions play in urges and behaviours, acceptance of one’s own contribution to current difficulties and the ability to tolerate the emotions that this self-reflection induces. Moreover, it has previously been proposed that mentalising is necessary for recovery from BPD and that it is one important mechanism of change that underlies the effectiveness of various treatment modalities [3, 106].

Furthermore, people with an adult preoccupied attachment relationship style may have learned that outward demonstrations of distress function to maintain proximity to their attachment figures [105]. Therefore, if people have been reinforced for behavioural displays of emotional instability, it makes sense that they would experience ambivalence for engaging in the effortful work needed to effect changes required for recovery. These attitudes, alongside deficits in the very mental capacities that are required to make gains in therapy and possible ambivalence about change, may coalesce to contribute to explaining why preoccupied attachment styles appear to hinder improvement. Therefore, it may be expected that long term therapy will be necessary to assist people with adult preoccupied attachment relationship styles to move towards recovery from BPD.

Dismissing the importance of attachment relationships was associated with a higher likelihood of being a non-responder to BPD treatment in terms of global functioning. This result is in direct contrast to previous research which reported that changes from ambivalent to avoidant attachment were associated with better outcomes [107], and that patients with a dismissing style demonstrated the largest global functioning gains after therapy [98]. One possible reason for dismissive attachment being associated with positive outcomes in previous research could be that changing from a preoccupied or ambivalent attachment style to a dismissive or avoidant attachment style is likely to be associated with reductions in abandonment sensitivity and increases in self-reliance, which in turn, could be associated with better functioning. Measuring change in attachment style across time was beyond the scope of this investigation. The present findings indicate that a dismissive attachment style at baseline was associated with higher likelihood of being less functional at 12 months. People with a dismissive attachment style possess a positive view of themselves that is not reliant on others; furthermore, they have been found to be low on emotional expressiveness, warmth and intimacy in close relationships [39]. Both of these factors could then lead to people with a dismissive attachment style being less inclined to need or maintain close relationships. This inclination could subsequently have negative impacts upon their global functioning since they may have less connected social lives and possibly less occupational opportunities due to a reluctance to develop relationships in the workplace.

A dismissive relational style could also be detrimental in the therapeutic relationship. People with dismissing attachment styles often have difficulty asking for help and are often resistant to accepting support when it is offered [33, 100]. They can also minimise the emotional impact their life experiences have had upon them and exclude their therapists from their lives [100, 108]. These factors could prevent the client from engaging or investing in therapy to the level needed to facilitate change, resulting in a poor response to psychotherapy. Alternatively, the clinician may be influenced to be less active in the treatment, in turn, giving less help when it may be needed, due to the dismissive patient’s interactional style and appearance of functionality due to a tendency to talk less, downplay distress and present as self-sufficient [99].

In the present investigation, a secure attachment at baseline did not contribute to non-response. Secure attachment is comprised of positive models of self and others, and associated with positive therapeutic alliances and outcomes [33]; therefore, it is an expected finding that a secure attachment style did not contribute to non-response in the current study. Furthermore, no association was found between fearful attachment style and non-response. Although this attachment style is common among people with BPD and may play a pivotal role in the development of the disorder, previous research has demonstrated an inconsistent relationship [33, 109]. Therefore, more investigation will be needed to further understand the role that fearful attachment style plays in regarding its contribution to non-response.

In order to understand the differential associations between attachment styles and BPD non-response, a consideration of attachment as an overarching concept that exists on a continuum from self to other reliance may be useful. Secure attachment represents a balance between self and other reliance and fearful attachment represents a conflict between self and other reliance, both of which were both found to have no association with non-response to psychotherapy. Contrastingly, dismissive attachment which represents an overreliance on the self and preoccupied attachment which represents an overreliance on others, were both found to be associated with non-response. These findings may demonstrate that to achieve wellness through psychotherapy, there needs to be a balance between self and other reliance. Put simply, at one end of the continuum, overreliance on others is undesirable for psychological health, and at the other end of the continuum, overreliance on self is disadvantageous for psychological health. One possible implication is that clinicians can further aid response to psychotherapy by facilitating this balance via responding less to their countertransferrential pulls in one way or the other, and to focus on an evenly hovering curious stance, reflecting rather than reacting, to what the patient needs to ensure this balance.

Self-criticism was hypothesised to contribute to non-response due its previous associations with negative outcomes [32, 47, 48, 110–113]. However, the results indicated no relationship between self-criticism and non-response. Blatt [47] noted that people high in perfectionistic self-criticism are often prone to developing psychopathology; yet, they can also be high achievers. Therefore, it is conceivable that in this sample, self-criticism could have served as motivation for participants to exert the effort needed to make improvements in therapy, which could have functioned to reduce the risk of non-response. Furthermore, self-criticism could be seen to be more fluid and more available to conscious awareness, rendering it more malleable compared to attachment styles. Therefore, it is possible that self-criticism can change faster during treatment and has less influence on psychotherapeutic outcomes.

An exploratory approach was taken to investigating which individual BPD criteria contributed to non-response. Three individual BPD symptoms were related to non-response. Firstly, anger was found to increase the risk of being a non-responder in terms of BPD symptom severity. In the context of BPD, anger has been related to early treatment termination, the exacerbation of interpersonal conflicts [114] and poor outcomes after therapy [115]. In the current sample, anger could have increased the risk of being a non-responder by contributing to interpersonal conflicts between the consumer and clinician, which could have led to negative countertransference, treatment resistance, or early termination.

Secondly, paranoia contributed to non-response in terms of global functioning. In the context of BPD paranoid thinking is common [116, 117] and has been found to be negatively correlated with functioning [116]. Being chronically suspicious of others could make it difficult to develop long lasting relationships or keep a job, and consequently, could lead to being less functional. Past research has noted that although symptomatic remission from BPD is common; recovery, typically defined as symptomatic remission and good global functioning, is much more difficult to attain [95, 96]. This novel finding could begin to explain why.

Lastly, relationship instability was found to reduce the risk of being a non-responder in terms of global functioning. Although counterintuitive at first glance, individuals with a higher rating of relational instability at baseline may need a stable relationship for the facilitation of functional recovery. People with BPD often experience emotional overwhelm and loss of mentalising capacities in the context of interpersonal crises [35, 118–120]. It may be that the participants who gave high endorsements on the criteria ‘relationship instability’ presented to emergency seeking help after experiencing distress due to not getting their relational needs met. This segment of the sample may have gone on to make functional gains in their relationship with their psychotherapist since long-term psychotherapy can provide a social environment in which a person with BPD can increase efficacy for better communication, maintaining connections and repairing ruptures. These new skills and experiences of self could have generalised, and therefore, increased the duration, quality and satisfaction of the relationships among their own social networks.

### Clinical impacts

The results of the present study indicate that the adult attachment relationship style a person with BPD brings to treatment contributes to their outcomes. It is therefore recommended that when working with people with BPD, their typical relationship style be assessed early on to inform the approach to care. Previous researchers have noted that people with a preoccupied attachment style experience more ruptures in the therapeutic alliance over time [121] and that their progress is dependent upon the therapist’s ability to tolerate chaos [33]. The findings reported here suggest adult attachment relational style, particularly in the presence of high anger, can impede progress. Similarly, a dismissive and paranoid relational style is also challenging to treatment. Future research is needed to track how these variables can influence treatment from session to session and how changes from one attachment style to another across the course of treatment can enhance response to psychotherapy.

### Limitations

Using the BPD symptom RCI is a reasonable estimate of expected clinically significant change versus non-change, however, we acknowledge that others have adopted different criteria (e.g., a GAF score of 71+). We thus used three estimates: BPD Symptom RCI, MHI-5 RCI, and GAF 71+. Although findings were slightly different from each approach, there were consistencies in the findings. It is also acknowledged that using an overall BPD RCI score as the categorical variable for the DV, then using single items from that BPD measure as IVs is a limitation. To address this limitation, the MHI-5 was also used as an IV with the same DVs (attachment, self-criticism and BPD symptoms) in a logistic regression to look for similarities in results and to determine the veracity of the findings of the model that used the BPD measure as both DV and IVs. Similar results did emerge in that the preoccupied attachment style was associated with a greater likelihood of not responding to treatment regarding both symptomology and psychological distress. Another statistical limitation is that the SE and SD were calculated from the current sample’s baseline data as opposed to obtaining these values from other published data. Although this is less conventional, other authors have also used the same method [85, 91, 92].

A methodological limitation of the present investigation is that treatments in the community over the 12-months were ’ad libitum’ i.e., allowed to naturalistically vary between patients based on their individual needs and were not controlled. We were thus unable to investigate specific treatment types, intensities or other characteristics that might be contributing to non-response. We were also unable to engage a large proportion of participants for follow up interviews. Drop out can be considered a type of non-response; therefore, being unable to collect data from the people who dropped out may have shed a different light on the outcomes of this study. Furthermore, it was beyond the scope of the current study to capture the intricacies of the interpersonal relationships between each consumer and clinician, including therapeutic alliance. However, studies that explore the interactions between client and clinician and their associated attachment styles, will be an important area of focus for future research. It is also acknowledged that the GAF was assessed at follow up only, which prevented our ability to measure change in functioning. The use of brief measures could have reduced reliability and validity; a more comprehensive measurement strategy including more detailed adult attachment interview methods would strengthen the study. Further research using different measurement tools is recommended.

## Conclusions

Adult attachment relationship styles were found to be related to the problem of non-response to psychotherapy for BPD. Beginning therapy with an adult preoccupied attachment relationship style in particular was associated with higher risk of being a non-responder in terms of BPD symptoms and psychological distress after 12 months. This points to the importance of early identification of attachment patterns among consumers and adjusting the treatment and interpersonal interactional styles to suit accordingly. Interestingly, a different pattern of results was found to explain non-response regarding global functioning. Having a dismissing relationship attachment style and high paranoia was associated with higher likelihood of being a non-responder, while higher endorsements of relationship instability was associated with lower likelihood of being a non-responder, in terms of functioning. These novel findings may begin to shed light on why the attainment of functionality seems more difficult to reach in comparison to symptomatic remission.
